# High Prevalence of Panton-Valentine Leukocidin (PVL) Genes in Nosocomial-Acquired *Staphylococcus aureus* Isolated from Tertiary Care Hospitals in Nepal

**DOI:** 10.1155/2014/790350

**Published:** 2014-06-18

**Authors:** Bidya Shrestha, Winny Singh, V. Samuel Raj, Bharat Mani Pokhrel, Tribhuban Mohan Mohapatra

**Affiliations:** ^1^Tri-Chandra Campus, Tribhuvan University, G.P.O. Box 3285, Kathmandu, Nepal; ^2^New Drug Discovery Research, Ranbaxy Research Laboratories, R & D III, Sector-18, Gurgaon, India; ^3^Institute of Medicine, Tribhuvan University, Kathmandu, Nepal; ^4^Institute of Medical Sciences, Banaras Hindu University, Varanasi, India

## Abstract

Methicillin-resistant *Staphylococcus aureus* (MRSA) carrying the important virulence determinant, Panton-Valentine leukocidin (PVL), is an emerging infectious pathogen associated with skin and soft tissue infections as well as life-threatening invasive diseases. In carrying out the first PVL prevalence study in Nepal, we screened 73 nosocomial isolates of *S. aureus* from 2 tertiary care Nepali hospitals and obtained an overall PVL-positivity rate of 35.6% among the hospital isolates: 26.1% of MRSA and 51.9% of methicillin sensitive *S. aureus* (MSSA) isolates were found to be positive for the PVL genes. PVL prevalence was not associated with a specific (i) infection site, (ii) age group, or (iii) hospital of origin. It was found to be positively associated with heterogeneous MRSA (73.3%) compared to homogeneous MRSA (3.2%) and MSSA (51.9%); negatively associated with multiresistant MRSA (22%) compared to nonmultiresistant MRSA (60%) and MSSA (51.9%); and positively associated with macrolide-streptogramin B resistance (93.8%) compared to macrolide-lincosamide-streptogramin B resistance (0%) and no-resistance (45.8%) types. Macrolide-streptogramin B resistance was confirmed by the presence of *msr*(A) gene. Restriction pattern analyses provided evidence to support the circulation of a limited number of clones of PVL-positive MRSA, arguing for the adaptability of these isolates to a hospital setting.

## 1. Introduction

First reported in 1932, Panton-Valentine leukocidin (PVL) is a potent cytotoxin and an important virulence factor of* Staphylococcus aureus*. The toxin causes tissue necrosis and selectively disrupts leukocyte membranes, thus leading to enhanced virulence. Comprising two exoproteins encoded by two contiguous, cotranscribed, phage-encoded genes* lukS-PV* and* lukF-PV* [1, 2], PVL has been epidemiologically associated with virulent and highly transmissible strains of* S. aureus*. PVL-carrying* S. aureus* strains can cause serious skin and soft tissue infections and life-threatening invasive diseases like necrotizing fasciitis [3], purpura fulminans [4], and necrotizing hemorrhagic pneumonia [5]. PVL-related infections exhibit a preference towards attacking skin and soft tissues [6] but when the infection spreads to the lungs, the resulting necrotizing pneumonia rapidly destroys lung tissue and it has been proven to be lethal in 75% of the cases [7].

PVL-positive* S. aureus*, as a rapidly emerging worldwide phenomenon, is exerting a powerful presence in the global public health landscape. Even though heightened surveillance and improved case recognition might have played a role in increased reporting of such strains, the rapid growth of PVL-carrying* S. aureus* nonetheless should not be undermined. In 2010, Shallcross and colleagues reported data from the United Kingdom where 20% of* S. aureus* isolates obtained from skin and soft tissue infections were characterized as PVL positive [8]. This is an alarming rise from the 1.6% prevalence rate of PVL-positive* S. aureus* reported in the country in 2005 [9]. Even within the timespan of one year between 2005 and 2006, Ellington et al. noted a 2-fold increase in PVL-positive* S. aureus* in England and Wales [10]. Comparatively, countries in western and central Africa boast a higher prevalence rate at 57%, raising valid concerns for the spread of virulent PVL-positive MRSA clones [11]. Along similar lines, data from across the globe paints a varied picture for the prevalence of PVL-positive* S. aureus*: 12.8% in China [12], 30% in Germany [13], 1.8% in Ireland [14], 0.9% in Korea [15], 11.6% in Singapore [16], 4% in Turkey [17], and a striking 97% in the United States [18, 19].

Albeit being a relatively recent phenomenon,* S. aureus* clones that combine PVL production with methicillin resistance are rapidly emerging. This global emergence and propagation of clones combining methicillin resistance and PVL toxin production necessitates commensurate counteraction with improved vigilance, enhanced case ascertainment, and effective management of PVL-related disease. Rightfully regarded as a “superbug,” methicillin-resistant* S. aureus* (MRSA) by itself is a prominent cause of nosocomial infections and has been exacting a substantial toll on healthcare resources worldwide [20, 21]. Acquisition of the PVL genes by MRSA therefore represents a challenging conundrum in disease management and infection control.

To date, PVL has emerged as a significant virulence factor of community-acquired MRSA (CA-MRSA) [22] with relatively rare incidence of the toxin in hospital-acquired MRSA (HA-MRSA) infections. We conducted this study to investigate PVL prevalence among hospital isolates of* S. aureus *in tertiary care facilities of Nepal. To the best of our knowledge, this report on PVL carriage and gene detection is the first of its kind in Nepal. In this study, we explore the following: the presence of PVL genes in both HA-MSSA and HA-MRSA, the association between PVL gene and methicillin susceptibility pattern, its linkage with macrolide, lincosamide, and streptogramin resistance pattern, and the epidemiologic molecular typing of PVL-positive* S. aureus *by PCR-RFLP.

## 2. Material and Methods

### 2.1. Clinical Isolates

A total of 404 consecutive, nonrepeat, nosocomial* S. aureus* isolates were obtained from Lalitpur-Based Hospital (*n* = 100) and Kathmandu-Based Hospital (*n* = 304) from November 2007 to June 2009. A nosocomial infection was defined as any infection acquired by a patient after 48 hours of hospital admission. Isolate identification was based on observed fermentation in oxidation-fermentation test and positivity for catalase, clumping factor, staphylocoagulase, DNase, staphytect plus, and mannitol fermentation. The isolates were categorized into four groups on the basis of infection sites (Table 1): body fluid, surgical site infection (SSI), lower respiratory tract infection (RTI), and urinary tract infection (UTI). Antibiotic susceptibility profiles were developed using penicillin, oxacillin, cefoxitin, erythromycin, clindamycin, cotrimoxazole, chloramphenicol, ciprofloxacin, rifampicin, gentamicin, and tetracycline (Oxoid, Japan) as recommended by Clinical and Laboratory Standards Institute (CLSI) 2007 [23]. However, for urinary isolates, erythromycin, ciprofloxacin, and chloramphenicol were replaced by novobiocin (to allow for the differentiation between* S. aureus* and* S. saprophyticus*), norfloxacin, and nitrofurantoin (Oxoid, Japan).

MSSA and MRSA were identified based on their respective sensitivity or resistance to both oxacillin and cefoxitin. Oxacillin disc diffusion test result was confirmed by minimum inhibitory concentration (MIC) using the* E* test and microbroth dilution. Methicillin resistance was further confirmed by the amplification of* mecA* gene by polymerase chain reaction and by the MRSA screen test for the detection of the protein PBP2a responsible for reduced affinity of MRSA to *β*-lactam antibiotics (Denka Seiken, Japan).

Heterogeneous MRSA was identified on the basis of (i) the presence of colonies inside the oxacillin and cefoxitin zones of inhibition in the disc diffusion test and inside the oxacillin strip inhibition zone in the* E* test and (ii) oxacillin MIC values <100 *µ*g/mL in* E* test and microbroth dilution test [24]. Also, on the basis of resistance to non-*β*-lactam antibiotics, MRSA isolates were grouped as multiresistant (resistant to ≥3) and nonmultiresistant (resistant to ≤2) MRSA, as described by Gosbell and colleagues in 2001 [25].

And finally, macrolide-lincosamide-streptogramin B (MLS_B_) and macrolide-streptogramin B (MS_B_) resistance types were assessed via* D* test using erythromycin and clindamycin [23]. Isolates resistant to erythromycin and susceptible to clindamycin were identified as MS_B_ resistance type, isolates resistant to both erythromycin and clindamycin as MLS_B_ resistance type, and isolates sensitive to both antibiotics as no-resistance type. MLS_B_ and MS_B_ resistance were confirmed by the amplification of* erm*(A),* erm*(B),* erm*(C), and* msr*(A) as described by Lina et al., 1999 [6]. MS_B_ resistance was supported by the detection of* msr*(A) gene.

### 2.2. PVL Genes Identification

From our pool of clinical isolates, 46 MRSA and 27 MSSA (total 73 isolates) were genetically analyzed for PVL positivity by screening for the presence of the adjacent, cotranscribed* luk-PV* genes, namely,* lukS-PV* and* lukF-PV*, which encode the Panton-Valentine leukocidin toxin. These 73 isolates were randomly selected to include homogenous MRSA, heterogeneous MRSA, and MSSA.* S. aureus* ATCC 29213 and* S. aureus* ATCC 49775 were used as negative and positive controls, respectively. The primers (Sigma-Aldrich, Germany) used for the coamplification of PVL genes* lukS-PV* and* lukF-PV* were* lukPV-*1 (5′-ATC ATT AGG TAA AAT GTC TGG ACA TGA TCC A) and* luk-PV-*2 (5′-GCA TCA ACT GTA TTG GAT AGC AAA AGC) [26]. Total DNA was isolated from the clinical isolates and used as template for PVL gene identification. The samples were denatured at 94°C for 3 minutes followed by 30 cycles of the following steps: denaturation at 94°C for 30 seconds, annealing at 55°C for 30 seconds, extension at 72°C for 1 minute, and final extension at 72°C for 5 minutes [6]. The images were visualized and analyzed in Gel Doc (Bio-Rad, USA) by using the Quantity One 1-D Analysis Software.

### 2.3. PCR-RFLP for* Coa* and* Spa*

From the study sample size of 73 clinical isolates, all the PVL-positive isolates were selected for PCR-RFLP, including 12 MRSA and 14 MSSA isolates (further described in Section 3). The* coa* and* spa* genes of these 26 PVL-positive isolates (12 MRSA and 14 MSSA) and additional 9 PVL-negative isolates (4 MRSA and 5 MSSA) were amplified using the primer pairs* coa*1 (5′-CGA GAC CAA GAT TCA ACA AG) and* coa*2 (5′-AAA GAA AAC CAC TCA CAT CAG T) and* spa*1 (5′-ATC TGG TGG CGT AAC ACC TG) and* spa*2 (5′-CGC TGC ACC TAA CGC TAA TG) (Sigma Aldrich, Germany; [27]).

**Figure 1:**
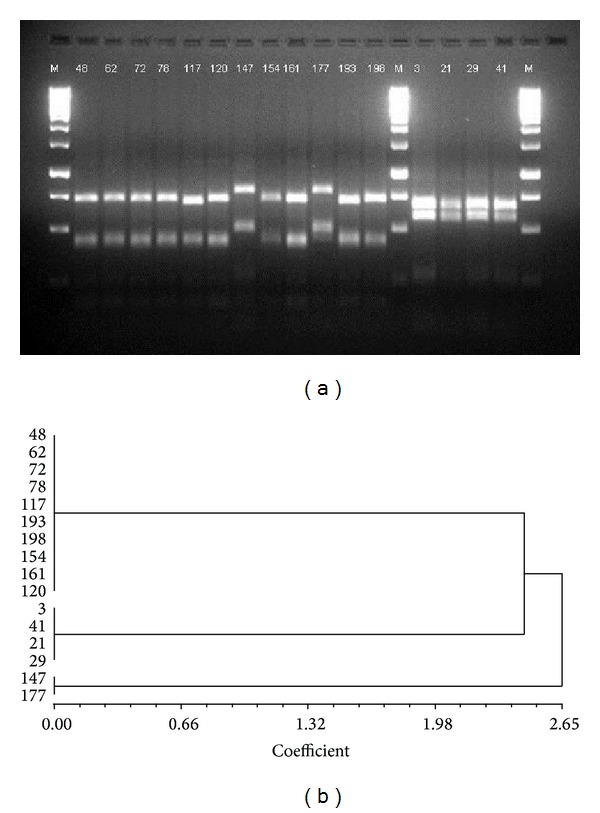
(a) PCR-RFLP of* coa* and* spa* amplified products of PVL-positive MRSA (12 isolates, 48–198) and PVL-negative MRSA (4 isolates, 3–41) digested by* Hae*II;* M* = 1 kb DNA ladder. PVL-negative MRSA isolates cluster under a single restriction pattern, while PVL-positive MRSA isolates demonstrate clustering under two distinct restriction types: type I (48–120, 154–161, and 193–198) and type II (147, 177). The isolates were obtained from two tertiary care hospitals. (b) Corresponding dendogram of the PCR-RFLP of PVL-positive and PVL-negative MRSA isolates.

**Figure 2:**
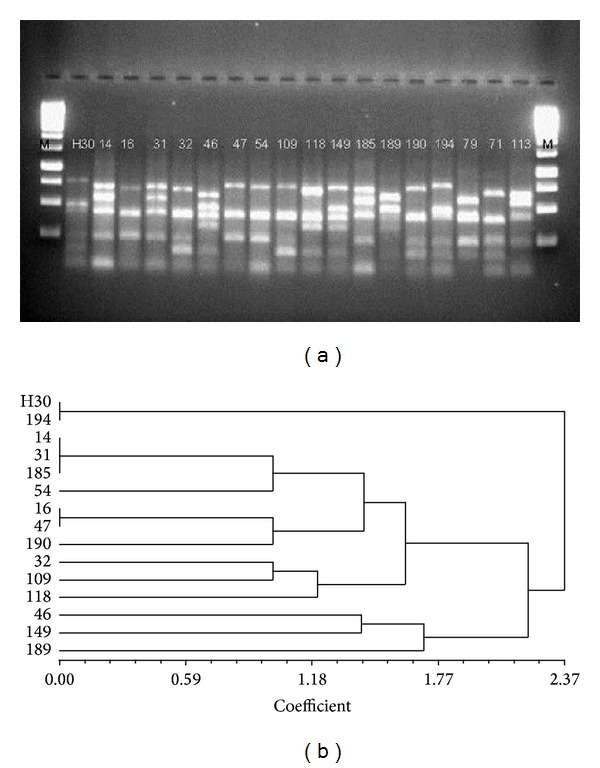
(a) PCR-RFLP of* coa* and* spa* amplified products of PVL-positive MSSA (14 isolates, 14–194) and PVL-negative MSSA (3 isolates, 79–113) digested by* Hae*II;* M* = 1 kb DNA ladder. PVL-positive MSSA isolates 16 and 47 cluster under one restriction type and isolates 14, 31, and 185 cluster under another. The remaining nine PVL-positive MSSA isolates and all three PVL-negative MSSA isolates exist as singletons. (b) Corresponding dendogram of the PCR-RFLP of PVL-positive and PVL-negative MSSA isolates.

PCR-RFLP was carried out using* Hae*II digestion of the amplified products. Unique PCR-RFLP patterns obtained following the electrophoresis of the* Hae*II digested PCR products were each designated as an independent restriction pattern (Figures 1 and 2).

### 2.4. Susceptibility Patterns of the PVL-Positive and PVL-Negative MRSA and MSSA

MIC of 12 different antibiotics was determined for 26 PVL-positive (12 MRSA and 14 MSSA) and 9 PVL-negative isolates (4 MRSA and 5 MSSA) by microbroth dilution [23]. The antibiotics included fusidic acid, tetracycline, Augmentin, clindamycin, levofloxacin, erythromycin, trimethoprim-sulfamethoxazole, gentamicin, vancomycin, linezolid, kanamycin, and rifampicin (Sigma-Aldrich, Germany). The clinical isolates were suspended in physiological saline (0.5 McFarland) using Densimat (Biomerieux, Italy) following which 100 *µ*L of each suspended isolate was dispensed into microtiter wells containing doubling dilutions of the 12 antibiotics in cation-adjusted Mueller Hinton Broth (Becton Dickinson and Company, USA). The microtiter plates were incubated at 37°C for 18 hrs and were read in reflected light.

Data was analyzed using the statistical software SPSS 11.5.

## 3. Results

The source sample of 404 hospital-acquired isolates consisted of 197 (48.8%) MRSA isolates, of which 148 (75.1%) were homogenous and 49 (24.9%) were heterogeneous [28]. As mentioned earlier, from the total sample of 404 clinical isolates, 73 isolates were randomly selected for further analysis, owing to resource limitations. Of these 73 isolates that comprised of 46 MRSA and 27 MSSA isolates, PVL gene was detected in 12 MRSA (26.1%) and 14 MSSA (51.9%) for a cumulative prevalence rate of 35.6%. As indicated in Table 1, the PVL carriage was high in samples from UTI (50%, 4/8), SSI (35.2%, 19/54), and lower RTI (22.2%, 2/9). However, cross tab calculation between UTI and SSI (*X* ^2^ = 0.655, *P* = 0.418) and SSI and lower RTI (*Xf* ^2^ = 0.583,  *P* = 0.445) showed that the difference in PVL prevalence in these sites did not reach statistical significance.

For our analysis, the patient population was grouped into 2 age groups: ≤30 years (*n* = 27) and ≥31 years (*n* = 46). The PVL-positivity rates were 37% for ≤30 age group and 34.8% for ≥31 age group (Table 1); these values were not found to be statistically significantly different (*X* ^2^ = 0.038, *P* = 0.846). We also did not find any association between PVL positivity and hospital of origin (Table 2). The PVL prevalence of 32.1% at KBH and of 45.0% at LBH did not meet the statistical criteria for significant difference (*X* ^2^ = 1.058, *P* = 0.304).

Upon conducting a pooled evaluation of isolates from both hospitals, PVL prevalence rates were found to be 73.3% in heterogeneous MRSA, 51.9% in MSSA, and 3.2% in homogeneous MRSA (Table 1). PVL occurrence was observed to be significantly associated with a heterogeneous methicillin resistance pattern compared to homogeneous MRSA and MSSA (*X* ^2^ = 26.59, *P* < 0.001). However, a pooled sample of both homogeneous and heterogeneous MRSA failed to show any correlation with PVL carriage when compared to MSSA (*X* ^2^ = 4.925, *P* = 0.026).

As mentioned previously, MRSA that was resistant to ≥3 non-*β*-lactam antibiotics was categorized as multiresistant MRSA (mMRSA) and that resistant to ≤2 non-*β*-lactam antibiotics was identified as nonmultiresistant MRSA (nmMRSA). The distribution of PVL carriage rates was 51.9% among MSSA, 60% among nmMRSA, and 22% among mMRSA (Table 1). Compared to MSSA and nmMRSA, mMRSA had lesser prevalence of PVL positivity (*X* ^2^ = 7.739, *P* = 0.021).

In assessing the putative relation between PVL prevalence and macrolide, lincosamide, and streptogramin resistance types, we found that none of the isolates with MLS_B_ resistance (*n* = 32) was positive for PVL. Among MS_B_ resistance types, PVL positivity was at 93.8% and among nonresistant isolates depicting susceptibility to both erythromycin and clindamycin, PVL carriage was at 45.8% (Table 1). We found that MS_B_ resistance types were significantly associated with PVL gene carriage when compared to MLS_B_ resistance (*X* ^2^ = 42.11, *P* < 0.001) and no-resistance types (*X* ^2^ = 9.689, *P* = 0.002). Isolates susceptible to erythromycin and clindamycin (no-resistance type) had 17.7 greater odds of being PVL negative as compared to MS_B_ resistance types (OR = 17.727, 95% CI: 2.0 to 156.5).

The MS_B_ resistance type was genotypically confirmed by the presence of* msr*(A) gene. The isolates harboring this gene depicted a strong association with PVL gene possession (*X* ^2^ = 12.049, *P* = 0.001). Compared to isolates carrying the* msr*(A) gene, no-resistance type isolates lacking this gene were 45.5 times more likely to be not positive for PVL (OR = 45.5, 95% CI: 3.5 to 594.7).

The MIC of the 26 PVL-positive and 7 PVL-negative isolates was determined by microbroth dilution method. No substantial difference could be noted in the resistance pattern between PVL-positive and PVL-negative* S. aureus *strains (both MRSA and MSSA), with the exception of tetracycline and clindamycin, to which PVL-positive and PVL-negative isolates responded differently. It was observed that while PVL-positive MRSA was sensitive to both clindamycin and tetracycline, PVL-negative MRSA was uniformly resistant to these antibiotics. Further, both PVL-positive and PVL-negative MSSA isolates were also sensitive to clindamycin and tetracycline.

With regard to restriction types identified by PCR-RFLP, PVL-positive MRSA, PVL-negative MRSA, PVL-positive MSSA, and PVL-negative MSSA isolates depicted two, one, eleven, and three restriction types, respectively (Figures 1 and 2). The majority of the PVL-positive MRSA isolates were clustered under one restriction type (10/12; DNA fragments length 400 bp and 750 bp) with the remaining two isolates being grouped under the second restriction type (2/12; DNA fragments length 500 bp and 800 bp). All four isolates of PVL-negative MRSA belonged to a single restriction type. For PVL-positive MSSA, two and three isolates were included under two distinct restriction types, while the remaining nine isolates and all three of the PVL-negative MSSA isolates existed as singletons within their unique restriction types (Figure 2).

## 4. Discussion

In the present day context, when microbial antibiotic resistance is progressively morphing into an intractable predicament, acquisition of the potent PVL gene by MRSA, which represents a rather difficult challenge in itself, would pave the way to an alarming healthcare paradox. In this paper, we present the findings from the first PVL prevalence study in Nepal, which includes (i) heretofore unidentified associations between PVL positivity and MS_B_ resistance, verified by* msr*(A) gene, (ii) PVL carriage rates specific to MRSA and MSSA isolates obtained from two premiere tertiary care hospitals, (iii) evaluation of putative predilection of PVL-positive* S. aureus* to a select patient profile, (iv) comparative assessment of PVL prevalence among heterogeneous MRSA-homogeneous MRSA-MSSA and among mMRSA-nmMRSA-MSSA, and (v) restriction pattern characterization of PVL-positive and PVL-negative hospital isolates.

In the current study, we report PVL gene carriage rate of 35.6% among nosocomial* S. aureus* isolates: 26.1% among MRSA and 51.9% among MSSA (Table 1). These numbers track along some of the highest reported PVL-positive* S. aureus* prevalence rates and should rightfully be regarded as a matter of grave concern. Further, restriction pattern analyses of PVL-positive MRSA and MSSA have revealed that, while PVL-positive MSSA can be grouped into eleven restriction types with a majority of the isolates existing as singletons (Figure 2), PVL-positive MRSA seamlessly clusters under two restriction types (Figure 1). This is an anticipated finding since MRSA isolates belong to a single clone, while MSSA isolates propagated from multiple clones. Such spread of a limited number of PVL-carrying MRSA clones suggests that, compared to their MSSA counterparts, these microbes are well-adapted to thrive in the hospital environment. The restriction type limitation would also follow from the fact that the MRSA isolates are hospital strains that cause infection in any susceptible host with the right combination of risk factors. Also, the restriction pattern clusters included isolates from both LBH and KBH, suggesting an interhospital transmission of pathogenic strains. These findings, in conjunction with the high PVL-positive* S. aureus* prevalence rates, warrant the immediate institution of effective infection control measures.

In addition to clone limitation, lack of any trend of higher PVL prevalence among a younger age group is another finding explained by the hospital-acquired nature of the PVL-positive* S. aureus* strains in the study. At prevalence rates of 37% for ≤30 years age group and 34.8% for ≥31 years age group (Table 1), the PVL-positive isolates fail to show any age-related predilection. Previous studies have shown a strong predisposition of PVL-positive* S. aureus* isolates for younger and previously healthy patients [14, 29]. In a 2008 Australian study by Munckhof et al., the authors observed a steady decline in PVL occurrence with increasing age, which they ascribed to age-associated strengthening of immunity and to the natural penchant of children and young adults to acquire PVL-positive* S. aureus* from skin contamination during playful and contact sports [29]. This increased likelihood of PVL-positive* S. aureus* to infect younger age groups, as seen in earlier studies, may be attributed to the fact that a majority of PVL-carrying* S. aureus* isolates from these studies were community-acquired. In contrast, our study exclusively investigated nosocomial isolates, which, regardless of age, can infect any patient presenting with the right risk factors for acquiring an infection originating in the hospital.

As with age, PVL prevalence also exhibited no association with infection site or hospital of origin. Although the highest numbers of PVL-carrying* S. aureus* isolates were recovered from UTI (50%), followed by SSI (35.2%) and lower RTI (22.2%), the prevalence rate difference between the sites did not reach statistical significance (Table 1). Along similar lines, the 45% carriage rate of PVL gene in LBH was not statistically significantly different from the 32.1% prevalence rate at KBH (Table 2). On the basis of vastly different occurrence of the PVL-positive organisms in different parts of the world, it appears that the PVL carriage depends largely on the geographical location and the organisms that are endemic in a particular locality.

Overall, PVL carriage rates were 51.9% (14/27) among MSSA, 73.3% (11/15) among heterogeneous MRSA, and 3.2% (1/31) among homogeneous MRSA (Table 1). Compared to MSSA and homogeneous MRSA, heterogeneous MRSA depicted a positive association with PVL carriage. However, when heterogeneous and homogeneous MRSA were pooled together into a single cohort of MRSA isolates and compared to MSSA, MRSA was found to have a negative association with PVL prevalence. The negative correlation between PVL and MRSA as a whole can be attributed to the 30 (96.8%) PVL-negative homogeneous MRSA isolates which would offset the PVL positivity of the 11 (73.3%) heterogeneous MRSA isolates. These findings suggest that, in the observed hospital settings, PVL positivity was associated with heterogeneous MRSA but not with homogeneous MRSA. Consequently, crude analyses of MSSA versus pooled MRSA strains could be potentially misleading; a thorough evaluation of PVL emergence should constitute a breakdown of MRSA into homogeneous and heterogeneous strains.

A phenotypic analysis of the clinical isolates based on their antibiotic profiles allowed for a grouping of the isolates into 3 categories, each of which revealed a relatively high PVL prevalence: 60% nmMRSA, 22% mMRSA, and 51.9% MSSA (Table 1). Munckhof and colleagues (2008) [29] report a similar rate of PVL positivity among their nmMRSA isolates (55%). However, their PVL prevalence rates of 2% among mMRSA and 16% among MSSA are much lower compared to this study.

Classifying the clinical isolates cohort on the basis of macrolide, lincosamide, and streptogramin resistance types, it was found that, while PVL positivity was 93.8% for MS_B_ and 45.8% for no-resistance types, none of the isolates with MLS_B_ resistance harbored the PVL gene (Table 1). This finding that isolates characterized as MS_B_ resistance type are more likely to depict PVL positivity compared to either MLS_B_ resistance or no-resistance type was verified by testing for the presence of* msr*(A) gene, which showed a positive association with PVL.

While it would have been ideal to carry out these molecular epidemiological analyses on the entire cohort of 404 isolates, the study needed to limit its sample size to 73 randomly selected isolates due to resource and monetary restrictions. Today's context of PVL diagnosis and epidemiology is constrained by the lack of a reliable phenotypic test for PVL positivity. Studies that focus on developing such novel methodologies for phenotypic characterization of PVL-positive bacterium, which would complement the currently available genetic test, will have substantial utility in promoting the early detection of these virulent microbes.

## 5. Conclusion

Our study has shown a relatively high prevalence rate of PVL positivity in HA-*S. aureus* isolates from two tertiary care hospitals of Nepal. PVL carriage was associated with heterogeneous MRSA and MS_B_ resistance type (confirmed by* msr*(A) gene positivity). Compared to MSSA and nmMRSA, mMRSA was significantly less likely to harbor the PVL genes. No association with age, site of infection, or hospital of origin was observed. Such an absence of predilection for a younger age group and the observed restriction pattern limitation of PVL-positive MRSA are in alignment with the fact that these were clinical isolates obtained from hospital-acquired infections. The relatively high rates of PVL presence among HA-MRSA and HA-MSSA isolates and the interhospital spread of strains support the need for system-wide implementation of patient safety and infection control initiatives.

## Figures and Tables

**Table 1:** PVL prevalence grouped according to infection site, age group, methicillin resistance, and macrolide, lincosamide, and streptogramin B resistance types.

		PVL-negative isolates (%)	PVL-positive isolates (%)
Infection site	Body fluid (*n* = 2)	1 (50.0%)	1 (50.0%)
	SSI (*n* = 54)	35 (64.8%)	19 (35.3%)
	Lower RTI (*n* = 9)	7 (77.8%)	2 (22.2%)
	UTI (*n* = 8)	4 (50.0%)	4 (50.0%)

Age group	≤30 years (*n* = 27)	17 (63.0%)	10 (37.0%)
	≥31 years (*n* = 46)	30 (65.2%)	16 (34.8%)

Methicillin resistance	MSSA (27)	13 (48.1%)	14 (51.9%)
	MRSA (46)	34 (73.9%)	12 (26.1%)
	(i) Heterogeneous MRSA (*n* = 15)	4 (26.7%)	11 (73.3%)
	(ii) Homogeneous MRSA (*n* = 31)	30 (96.8%)	1 (3.2%)
	(i) nmMRSA (*n* = 5)	2 (40.0%)	3 (60.0%)
	(ii) mMRSA (*n* = 41)	32 (78.0%)	9 (22.0%)

Macrolide, lincosamide, and streptogramin B resistance	No resistance (*n* = 24)	13 (54.2%)	11 (45.8%)
	MS_B_ (*n* = 16)	1 (6.2%)	15 (93.8%)
	MLS_B_ (*n* = 32)	32 (100.0%)	0 (0.0%)

SSI: surgical site infection; RTI: respiratory tract infection; UTI: urinary tract infection; nmMRSA: nonmultiresistant MRSA; mMRSA: multiresistant MRSA; MS_B_: macrolide-streptogramin B resistance; MLS_B_: macrolide-lincosamide-streptogramin B resistance.

**Table 2:** Hospital-specific PVL prevalence rates and methicillin resistance in *S. aureus*.

Strains (*n*)	Lalitpur Hospital			Kathmandu Hospital			Total
	PVL Pos (*n*)	PVL Neg (*n*)	PVL Pos %	PVL Pos (*n*)	PVL Neg (*n*)	PVL Pos %	PVL Pos %
MSSA (27)	4	3	57.1%	10	10	50.0%	51.9% *(14/27) *
Homo-MRSA (31)	0	7	38.5%^a^	1	23	21.2%^b^	26.1% *(12/46) *
Hetero-MRSA (15)	5	1		6	3		

Total (73)	9	11	45.0%	17	36	32.1%	35.6% *(26/73) *

^a^Total PVL-positive prevalence rate in Lalitpur Hospital.

^b^Total PVL-positive prevalence rate in Kathmandu Hospital.

Hetero: heterogeneous; Homo: homogeneous; Pos: positive; Neg: negative.
